# Molecular subtypes of breast cancer in relation to paclitaxel response and outcomes in women with metastatic disease: results from CALGB 9342

**DOI:** 10.1186/bcr1622

**Published:** 2006-11-27

**Authors:** Lyndsay N Harris, Gloria Broadwater, Nancy U Lin, Alexander Miron, Stuart J Schnitt, David Cowan, Jonathan Lara, Ira Bleiweiss, Donald Berry, Matthew Ellis, Daniel F Hayes, Eric P Winer, Lynn Dressler

**Affiliations:** 1 Dana-Farber Cancer Institute, 44 Binney Street, Boston, Massachusetts 02115, USA; 2 Duke University Medical Center, 2301 Erwin Road, Durham, North Carolina 27710, USA; 3 Beth Israel Deaconess Medical Center, 330 Brookline Avenue, Boston, Massachusetts 02215, USA; 4 University of North Carolina, 250 East Franklin Street, Chapel Hill, North Carolina 27514, USA; 5 St. Barnabas Medical Center, 94 Old Short Hills Road, Livingston, New Jersey 07039, USA; 6 Mount Sinai School of Medicine, One Gustave L. Levy Place, New York, New York 10029, USA; 7 MD Anderson Cancer Center, 1515 Holcolmb Boulevard, Houston, Texas 77030, USA; 8 Washington University Medical Center, 660 S. Euclid Avenue, St. Louis, Missouri 63110, USA; 9 University of Michigan Comprehensive Cancer Center, 1500 E. Medical Center Drive, Ann Arbor, Michigan 48109, USA

## Abstract

**Introduction:**

The response to paclitaxel varies widely in metastatic breast cancer. We analyzed data from CALGB 9342, which tested three doses of paclitaxel in women with advanced disease, to determine whether response and outcomes differed according to HER2, hormone receptor, and p53 status.

**Methods:**

Among 474 women randomly assigned to paclitaxel at a dose of 175, 210, or 250 mg/m^2^, adequate primary tumor tissue was available from 175. Immunohistochemistry with two antibodies and fluorescence *in situ *hybridization were performed to evaluate HER2 status; p53 status was determined by immunohistochemistry and sequencing. Hormone receptor status was obtained from pathology reports.

**Results:**

Objective response rate was not associated with HER2 or p53 status. There was a trend toward a shorter median time to treatment failure among women with HER2-positive tumors (2.3 versus 4.2 months; *P *= 0.067). HER2 status was not related to overall survival (OS). Hormone receptor expression was not associated with differences in response but was associated with longer OS (*P *= 0.003). In contrast, women with p53 over-expression had significantly shorter OS than those without p53 over-expression (11.5 versus 14.4 months; *P *= 0.002). In addition, triple negative tumors were more frequent in African-American than in Caucasian patients, and were associated with a significant reduction in OS (8.7 versus 12.9 months; *P *= 0.008).

**Conclusion:**

None of the biomarkers was predictive of treatment response in women with metastatic breast cancer; however, survival differed according to hormone receptor and p53 status. Triple negative tumors were more frequent in African-American patients and were associated with a shorter survival.

## Introduction

The taxanes (paclitaxel and docetaxel) are among the most active drugs for treatment of breast cancer and are an important component of treatment in the neoadjuvant, adjuvant, and metastatic settings. These drugs act, at least in part, by stabilizing microtubules and inducing G_2_/M arrest, with subsequent apoptosis in malignant cells. Paclitaxel, the first taxane that was developed, has substantial antitumor activity. As a result, it has become the standard of care as first-line treatment in the metastatic setting, and it is indicated for the treatment of high-risk, early-stage breast cancer, in addition to anthracycline-based therapy [1-8]. However, little is known about the mechanisms of sensitivity and resistance to taxanes. Hence, an important therapeutic goal is to identify molecular predictors of a response to taxane therapy.

Human epidermal growth factor receptor (HER)2 (ErbB2) is over-expressed in 25% to 30% of human breast cancers and is associated with reduced disease-free survival and overall survival (OS) [9]. Data from Cancer and Leukemia Group B (CALGB) trial 8869/8541 [10] and National Surgical Adjuvant Breast and Bowel Project (NSABP) trial B11 [11] indicate an improved response to doxorubicin-containing regimens in patients whose tumors over-express HER2. Data on the association between this growth factor and taxane sensitivity are limited and conflicting. In some experimentalsystems, transfection of *HER2 *leads to paclitaxel resistance by blocking activation of Cdc2, which is required for paclitaxel-induced apoptosis [12,13]. HER2 signaling also activates Akt, providing a survival signal through that pathway [14]. In contrast, HER2 activation may increase sensitivity to paclitaxel by abrogation of the G_1_/S checkpoint through p27, allowing progression to G_2_/M [15].

Clinical studies of HER2 tumor levels in relation to the response to taxanes have also yielded conflicting results. Seidman and collaborators [16] reported an association between HER2 over-expression and an improved response to taxanes in a retrospective analysis of patients with breast cancer who had been treated in several clinical trials. Other studies have found no association with treatment response [17,18], resistance to paclitaxel [19], or an improved response in patients with HER2-positive tumors [20-22]. These differing results may be due to differences in patient selection, study design and sample size, and variation in the methods used to determine HER2 status.

Mutations in the tumor suppressor gene p53 are present in 18% to 25% of primary breast carcinomas [23,24]. In general, missense mutations lead to nuclear accumulation of p53, but the mutant protein is not capable of normal p53 function. Mutations in p53 may increase sensitivity to paclitaxel by abrogating the G_1 _checkpoint, allowing cells to progress to G_2_/M, as suggested by *in vitro *models [25]. In addition, expression of MAP4 (microtubule-associated protein 4), which occurs when p53 is transcriptionally silent, stabilizes polymerized microtubules and increases sensitivity to paclitaxel [26,27]. Alternatively, p53 mutations may lead to taxane resistance, because mutant p53 cannot upregulate expression of Bax, which participates in apoptosis [28]. Finally, specific mutations in p53 have been shown to disrupt spindle checkpoint control, potentially increasing resistance to taxane-induced damage [29].

Newer approaches to molecular classification of breast cancer have identified three distinct subclasses with both biologic and prognostic significance. These subclasses, defined by classic biomarkers, are estrogen receptor (ER) and/or progesterone receptor (PR) positive tumors, *HER2*-amplified tumors, and ER/PR/HER2-negative tumors. The three subtypes have been reproducibly identified by gene expression profiling in multiple breast cancer cohorts and exhibit consistent prognostic significance [30-34]. In addition, it was recently reported that African-American women are more likely to have the 'triple negative' breast cancer phenotype [35,36]. These tumors lack expression of ER, PR, and HER2; they express basal keratins and myoepithelial markers; and they are associated with worse outcome in early-stage breast cancer [34,37].

In CALGB 9342, women with advanced breast cancer were randomly assigned to paclitaxel, given alone as first-line or second-line chemotherapy, at one of three dose levels [8]. We conducted a biomarker companion study to determine whether HER2 status (measured by immunohistochemistry [IHC] and fluorescent *in situ *hybridization [FISH]), hormone receptor status, p53 status (measured by IHC and sequencing), or a combination of these markers predicted clinical outcomes in this cohort of patients with metastatic breast cancer. We also determined the frequency of molecular markers in African-American women and examined whether race influenced outcomes.

## Materials and methods

### Trial design

Women with histologically documented advanced breast cancer (stage IV or inoperable) who had measurable disease, an Eastern Cooperative Oncology Group performance status of 0, 1, or 2, a life expectancy of at least 12 weeks, and adequate end-organ function were eligible for enrollment in CALGB 9342. Patients were required not to have received more than one prior chemotherapy regimen for metastatic disease, and concurrent hormonal therapy was not permitted. Women were randomly assigned to receive paclitaxel at one of three doses – 175 mg/m^2^, 210 mg/m^2^, or 250 mg/m^2 ^– administered as a 3-hour infusion every 3 weeks. Standard staging studies were repeated after every three cycles. Patients were treated until progression of disease or toxicity required discontinuation of paclitaxel.

The study was approved by the institutional review boards of the participating centers, and all patients provided written informed consent. Of the 474 women who were enrolled, 469 received at least one cycle of paclitaxel as specified by the study protocol. A detailed description of the study design and results has previously been published [8].

### Objective response criteria

Response to paclitaxel was assessed radiographically every three cycles. A response was documented if radiographic studies showed a complete response, defined as the disappearance of all lesions, or a partial response, defined as at least a 50% reduction in the sum of the products of bidimensional measurements for all lesions, with improvement or no change in any nonmeasurable lesions. In the case of patients for whom response data were not available, those who were known to have had excessive toxicity or to have died early were included in the analysis as nonresponders. Patients for whom no follow-up information was available were excluded from the analysis.

### Hormone receptor status

Information on hormone receptor status was obtained from pathology reports.

Block collection and evaluation

Paraffin-embedded tumor tissue blocks were available from 201 (42%) of the 474 patients. For the other 273 patients, blocks were not available because they had been discarded as per institutional policy, because the resources for block collection were inadequate, or because the patient had died and the local institutional review board would not allow release of the tissue.

All samples were reviewed by a designated pathologist (IB). Sections stained with hematoxylin and eosin were evaluated for histologic grade and nuclear grade. Corresponding blocks that passed quality assurance were sent to the appropriate laboratory for analyses of HER2 and p53 status. Technicians were blinded as to outcome, race, and all other aspects of the clinical data. Because conflicting findings in previous studies of HER2 status and the response to paclitaxel may have been due to assay differences, three methods of measuring HER2 were used, and the level of agreement among them was determined.

### Immunohistochemistry studies of HER2 and p53

Analyses were conducted on formalin-fixed, paraffin-embedded specimens of tissue from the primary breast tumor, a lymph node metastasis, or a distant metastasis. Only one sample per patient was selected for the final analysis, with the sample from the primary site being used when available (90% of patients).

Immunostaining for HER2/*neu *expression was performed with the use of monoclonal antibody CB11 (Biogenex, San Ramon, CA, USA) and the Vector Avidin Biotin Complex (ABC) kit (Vector Laboratories, Burlingame, CA, USA), in accordance with the manufacturer's guidelines. After development with SG chromogen (Vector Laboratories), sections were counter-stained with nuclear fast red. A positive result was defined as staining of moderate to strong intensity in at least 10% of the invasive carcinoma cells.

HER2 immunostaining was also performed with the HercepTest (Dako Corp., Carpinteria, CA, USA), in accordance with the manufacturer's guidelines. A positive result was defined as a staining score of 3+, with complete membrane staining of more than 10% of tumor cells.

Expression of p53 was evaluated with the use of the D07 antibody (Oncogene Science, Cambridge, MA, USA) and the Vector ABC kit, in accordance with the manufacturer's guidelines. After incubation overnight with the D07 antibody, sections were developed with diaminobenzidine as the chromogen and counter-stained with hematoxylin. A positive result was defined as distinct nuclear staining in at least 10% of the invasive carcinoma cells.

### Fluorescence in situ hybridization analysis of HER2

*HER2 *gene amplification was performed with the use of the Vysis PathVysion kit (Vysis, Inc., Downers Grove, IL, USA), in accordance with the manufacturer's guidelines. Specimens were considered amplified if the ratio of *HER2 *to CEP17 signal was greater than or equal to 2.0.

### p53 Mutation analysis

DNA was extracted from four 4 μmol/l formalin-fixed, paraffin-embedded tissue sections with macrodissection of epithelial elements away from stromal tissue (TN, AM, LH). After solubilization with xylene and overnight digestion of the tissue in proteinase K, DNA was isolated with the use of the Purgene kit (Gentra Systems, Minneapolis, MN, USA). After amplification, polymerase chain reaction products were purified, and terminator-based sequencing kits with Big Dye technology (Perkin-Elmer, Wellesley, MA, USA) were used to determine the sequence of each of the p53 coding exons (exons 2 through 10). The presence of mutations was ascertained using the Sequencher program (Gene Codes Corp., Ann Arbor, MI, USA). Mutations were confirmed by repeat sequencing of all exons. Identified mutations were compared with the p53 mutation database maintained at Hôpital Necker-Enfants Malades in Paris, France [38].

### Statistical methods

Each specimen was assigned a unique laboratory identification number. Laboratory investigators were unaware of patient identity and outcome data. Results were analyzed by the statistical center at CALGB (GB and DB).

The initial target for accrual was 332 patients (70% of the overall study population), with a goal being to evaluate the dose response to paclitaxel, dichotomized by biomarker status. Because the response rate was equivalent among the three groups in the overall study, and because the number of tissue blocks collected was less than anticipated, the three dose levels were combined for the current analysis. A dichotomous variable was generated for each biomarker (positive or negative).

Cohen's kappa statistic was used to measure the level of agreement between HER2 assay methods. We used a test of proportions to compare patient characteristics that were dichotomous. For patient characteristics collected as continuous variables, we compared median values, using the Wilcoxon rank-sum test. Response rates were compared on the basis of categorization of clinical variables and HER2 and p53 status, with the use of a test of proportions. Time to treatment failure (TTF) was measured as the time from study entry to the first locoregional recurrence, first distant metastasis, or death due to any cause; data were censored for patients without events at the last follow-up date. OS was measured as the time from study entry to death, with data censored at the last follow up. Time to event and Kaplan-Meier curves were compared using the log-rank test. Hazard ratios and their 95% confidence intervals (CIs) were calculated for multivariate Cox proportional hazards modeling. The significance of variables in multivariate Cox models was determined using the χ^2 ^test. All statistical tests were two sided.

## Results

### Baseline and follow-up characteristics of the patients

Paraffin-embedded tissue blocks were available for 201 of the 474 patients enrolled in CALGB 9342. Blocks from 175 patients passed quality control. A total of 165 patients with at least one biomarker measurement are included in this companion study. The median follow-up time was 8.3 years among patients with at least one biomarker measurement and 8.5 years for the remaining study population (Table 1). The only significant difference between the two groups was the shorter median disease-free interval among patients with biomarker measurements (19 versus 31 months; *P *= 0.0003). Because blocks are often discarded after 10 years, patients with available blocks would, by definition, have a shorter interval between diagnosis and metastasis.

### Hormone receptor, HER2, and p53 status

Information on hormone receptor status was available for 148 of the 201 patients from whom tissue blocks were collected. Of these 148 patients, 74 (50%) had tumors that were reported to be hormone receptor positive.

The CB11 assay of HER2 was informative in 162 cases, the HercepTest in 158 cases, and FISH in 152 cases. The results of these assays are summarized in Table 2 and their sensitivity and specificity in Table 3.

The D07 antibody assay for p53 protein was informative in 150 cases; 57 tumors (38%) exhibited evidence of p53 protein over-expression by this method.

### p53 Mutations

Sequencing of exons 4 to 11 of the p53 gene was informative in the 152 cases in which we were able to extract adequate DNA from the tumor specimen; 51 mutations were found in 44 patients (29%). Tumors from six patients contained more than one mutation in p53; in the remainder a single mutation was identified. Approximately half were missense mutations, and 31% were categorized as 'other' (in intron sequences). The remaining mutations were classified as splice mutations (9%) or as silent, nonsense, or frameshift mutations (4% each). The largest proportion of mutations was found in either intron 7 (27%) or exon 5 (22%). Mutations in exons 7 and exon 8 accounted for 12% and 18% of the identified mutations, respectively. Mutations in exons 4 and 10 were rare, and there were no mutations in exon 9. Only two tumors were found to have class II p53 mutations, which have been postulated to be associated with defects in spindle checkpoint control.

Because not all mutations in p53 lead to IHC evidence of p53 over-expression, we assessed the sensitivity and specificity of IHC for detecting p53 mutations that had been identified by sequencing. We found that IHC had a sensitivity of 63% and a specificity of 73%. Because previous studies have shown that over-expression of p53 is highly correlated with missense mutations [39], we evaluated the level of agreement between IHC evidence of p53 over-expression and the presence of a missense mutation in p53. IHC detected the majority of missense mutations identified by sequence analysis, with a sensitivity of 90%. However, the specificity was 72%, reflecting the fact that not all mutations detected by sequencing were detected by IHC.

### Treatment response

The rate of response to paclitaxel did not differ significantly on the basis of HER2 or p53 status (Table 4). The response rate was 23% among women with HER2-positive tumors and 24% among those with HER2-negative tumors (*P *= 0.96); the respective response rates for tumors with and for those without p53 over-expression were 23% and 21% (*P *= 0.79). Lower response rates were observed in patients aged 50 years or less (14% versus 29%) and a HER2 score of 0 to 1+ (18% versus 35%); however, only age was significantly associated with TTF (*P *= 0.045) after adjustment for ER/PR status and HER2 status (Table 5).

### Outcomes

**Figure 1:**
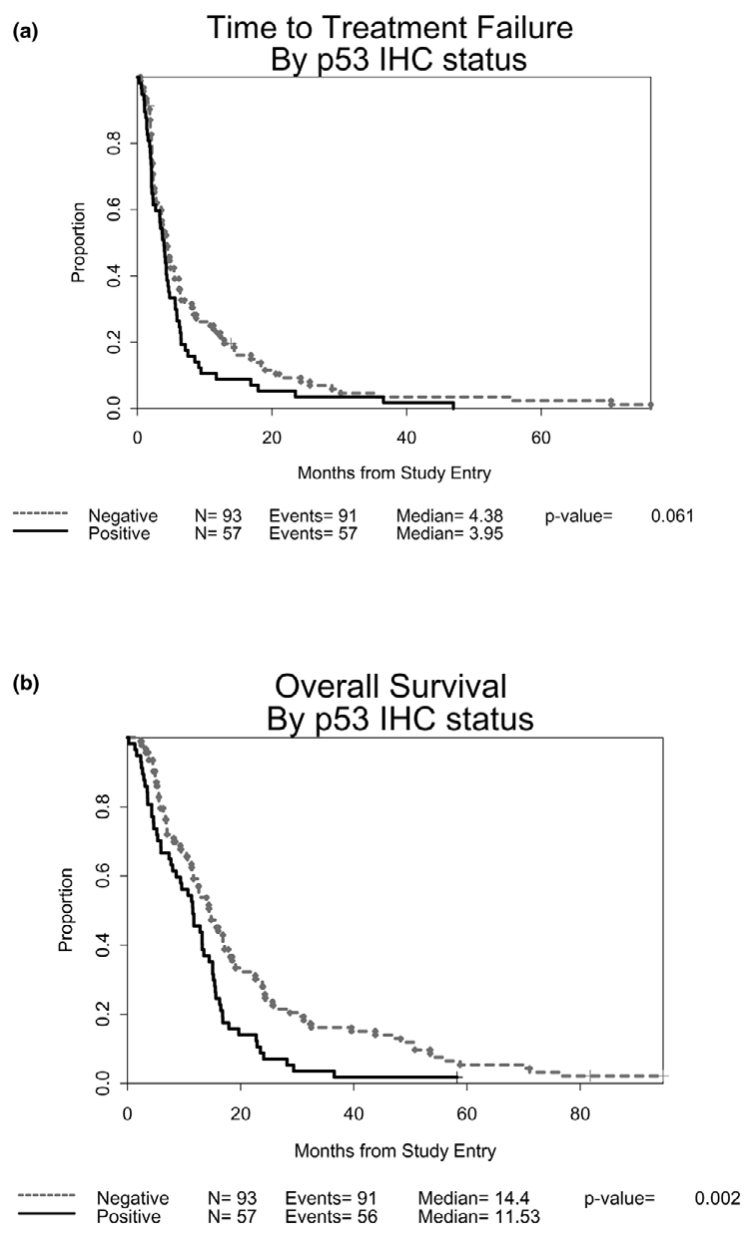
Time to treatment failure and overall survival according to p53 status, as assessed by immunochemistry. **(a) **Time to treatment failure and **(b) **overall survival. Patients were classified as p53 positive (solid line) or p53 negative (dashed line) as determined by immunochemistry with D07 antibody. A positive case is defined as ≥10% positive, localization of nuclear or nuclear and cytoplasmic, and intensity of stain weak, moderate, or intense. IHC, immunohistochemistry.

There was no meaningful difference in TTF between tumors with and without p53 over-expression (median time: 4.0 months versus 4.4 months; *P *= 0.061); however, OS was significantly reduced in this group of patients (11.5 months versus 14.4 months; *P *= 0.002; Figure 1). There was a trend toward a shorter TTF among patients who were HER2 positive than among those who were HER negative (2.3 months versus 4.2 months; *P *= 0.067), but HER2 status was not associated with statistically significant differences in OS (Table 6).

Although TTF did not differ by ER status (*P *= 0.13) in the biomarker subset, OS was significantly better in this group of ER-positive tumors (*P *= 0.003). This observation was also true of ER status in the entire cohort of patients from CALGB 9342, in which TTF did not differ by ER status (*P *= 0.27), whereas ER positivity was associated with more favorable OS (*P *= 0.0003).

To determine whether particular p53 mutations predicted a worse outcome, we grouped missense mutations into nonoverlapping categories, according to the methodology of Alsner and coworkers [24]. However, the number of patients in each subgroup was too small to make meaningful comparisons (nine with mutations in direct DNA contact or zinc binding residues, 10 with mutations in conserved domains, and 18 with mutations outside conserved domains).

### Triple-negative phenotype

**Figure 2:**
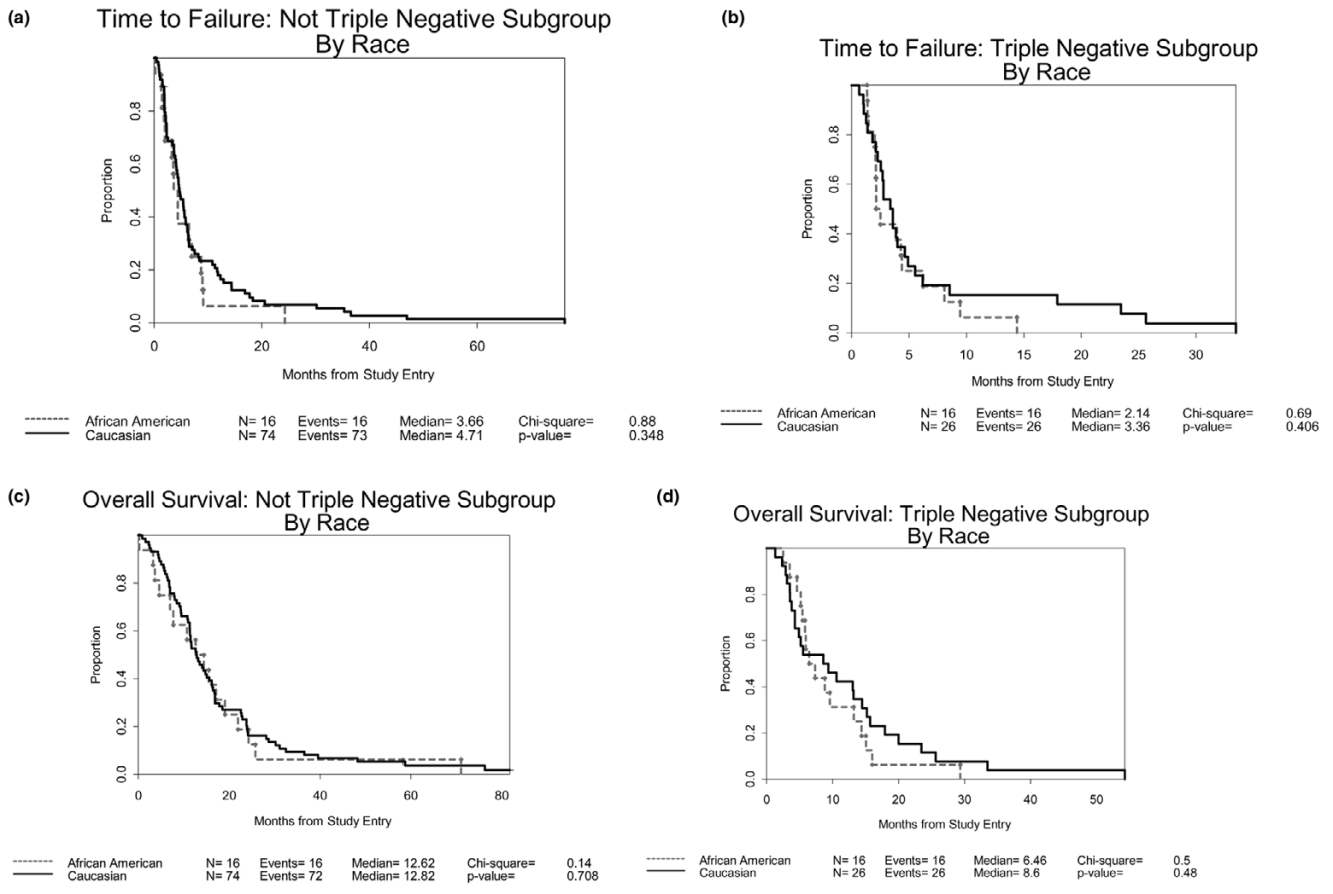
Time to treatment failure and overall survival for triple-negative subgroup, by race. Time to treatment failure: **(a) **not triple negative and **(b) **triple negative. Overall survival: **(c) **not triple negative and **(d) **triple negative. Patients were classified by race (African-American [dashed line] or Caucasian [solid line]) and divided into subsets based on triple-negative status. Exploratory analysis to investigate the interaction of triple negative status and race.

Of the 136 patients in this study for whom complete biomarker data were available, 44 had tumors that were found to carry the triple-negative phenotype (ER negative, PR negative, and HER2 negative). There was a higher proportion of triple-negative tumors with over-expression of p53 on IHC, but this result did not reach statistical significance (53% versus 36%; *P *= 0.088). We conducted an exploratory analysis to determine the objective response rate, TTF, and OS in patients with the triple-negative phenotype. We found that neither the response rate nor TTF differed in the triple-negative subgroup as compared with all other patients (response rate: 26% versus 23%, *P *= 0.70; TTF: 2.8 months versus 4.5 months, *P *= 0.092). However, the triple-negative phenotype was associated with a significant decrement in OS (8.6 months versus 12.8 months; *P *= 0.008; Figure 2). The results were similar when FISH was used to determine HER2 negativity in this subgroup (8.8 months versus 11.7 months; *P *= 0.038).

### Outcomes and biomarkers according to race

A total of 105 (22%) of the participants in CALGB 9342 identified themselves as African-American. An exploratory analysis showed that the response rate and TTF were similar in African-American women and Caucasian women. However, the median OS was significantly shorter among African-American women (10.1 months versus 13.1 months; *P *= 0.0005); the difference persisted in a multivariate analysis (hazard ratio 1.44, 95% CI 1.13 to 1.84).

The proportion of African-American women in the subset of patients with biomarker data (20.6%; *n *= 34) was similar to the proportion in the overall group. Tumors were HER2 positive, according to the CB11 assay, in 9% of African-American women, as compared with 22% of Caucasian women (*P *= 0.08). The percentage of African-American women presenting with tumors that were negative for ER, PR, and HER2 expression was more than twice that of Caucasian women (47% versus 21%; *P *= 0.003; Table 7). Both TTF and OS were significantly worse among African-American women than among Caucasian women (*P *= 0.038 and *P *= 0.045, respectively), a difference that persisted in a multivariate analysis (hazard ratio 1.44, 95% CI 1.13 to 1.84). However, when evaluating disease-free survival and OS in triple-negative tumors, survival did not differ by race, suggesting that the negative outcome of African-American women in this cohort is attributable to the greater proportion of triple-negative tumors and not other race-related variables (Figure 2). Of note, there were no significant differences between the proportions of African-American women and Caucasian women with p53 mutations as assessed by IHC (41% and 38%, respectively; *P *= 0.78) or by sequencing (32% and 26%, *P *= 0.48).

## Discussion

We evaluated the relationship among tumor biomarkers, treatment response, and outcomes in a subset of patients with available tumor blocks who were enrolled in a large, prospective, randomized trial of single-agent paclitaxel given as first-line or second-line therapy for metastatic breast cancer. Neither HER2 status nor p53 status significantly affected the treatment response or TTF when HER2 over-expression was scored as 3+ on the HercepTest or FISH positive. Although p53 status and ER status were not predictive of a benefit from therapy, measured by response or TTF, they were both important variables in determining OS in this cohort. Thus, it appears that p53 and ER status behaved as prognostic factors in this study, but they did not add predictive value within the context of response to paclitaxel. Although not surprising, this observation underlines the importance of separating response to therapy from the underlying biology, a distinction first made by McShane and coworkers [40]. In addition, our study provides support for the use of classification systems that include ER, PR, and HER2 status, because the outcomes according to these biomarkers in our cohort are consistent with published data [30,34]. Furthermore, racial differences in the pattern of tumor subtypes are also seen, a finding that translates into differences in survival, even in the metastatic setting.

Other studies have examined response to taxanes based on HER2 status. Seidman and coworkers [16] reported a higher rate of response to paclitaxel in patients with HER2-positive tumors; however, this association was seen only when HER2 status was assessed with the use of the 4D5 antibody, and not when a rabbit polyclonal antibody (pAb-1) was used to determine HER2 status. The authors reported a low level of agreement between the results of the 4D5 assay and an assay with pAb-1. The monoclonal antibody CB11 and the polyclonal antibody used in the HercepTest are known to target different epitopes of HER2 and appear to vary in the specificity of their results. Therefore, in the present study we evaluated the correlation among three methods of measuring HER2 status that have been approved by the US Food and Drug Administration. We found that the results of the CB11 assay, the HercepTest, and FISH were reasonably concordant, with the highest level of agreement between the CB11 assay and FISH.

No association was seen between the rate of response to paclitaxel and HER2 status as assessed by any method. In contrast to our findings, Konecny and coworkers [21] reported a statistically significant increase in response rate to epirubicin and paclitaxel, but not to epirubicin and cyclophosphamide, in women with HER2-positive tumors, as assessed by FISH. One possible explanation for these conflicting results is that the patient populations were not entirely comparable, because all patients in the study reported by Konecny and coworkers received treatment as first-line therapy, whereas in CALGB 9342 paclitaxel was given as either first-line or second-line therapy for metastatic disease. In addition, the treatment regimens were not entirely comparable. There may be an advantage to combining an anthracycline with a taxane in women with HER2-positive tumors; if so, epirubicin plus paclitaxel may be a particularly effective regimen against HER2-positive breast cancer, whereas HER2 status may not influence the likelihood of a response to paclitaxel given alone. Furthermore, FISH techniques differed between these studies and might have influenced the findings. Finally, our sample was relatively small, although the identical response rates in women with HER2-positive tumors and those with HER2-negative tumors suggests that a substantial difference in response rates would be unlikely in a larger sample.

Our data showed that hormone receptor status is important as a prognostic factor in this study but was not predictive of clinical response or TTF after paclitaxel therapy. Conflicting data have been reported regarding the role of ER in predicting benefit from taxane therapy. In CALGB 9344, a subgroup analysis demonstrated that the addition of paclitaxel in the adjuvant setting was more beneficial in women with ER/PR-negative tumors than in those with ER/PR-positive tumors [1]. This observation is supported by a combined analysis conducted by Henderson and coworkers [41], which demonstrated a strong relationship between ER/PR negativity and a benefit from chemotherapy. However, the results of the NSABP B-28 trial [2] and the Breast Cancer International Research Group (BCIRG) 001 trial [42] do not support this finding. Our study suggests that differences in survival as a result of hormone receptor status need to be considered in analyses of trials in the metastatic setting, even among patient populations that have previously received hormonal therapy.

We further evaluated the relationship among p53 status, race, and outcomes in this cohort. We found that the presence of p53 over-expression, as detected by IHC, was not predictive of the response to paclitaxel but was associated with significantly diminished OS. There are two possible explanations for these findings. First, mutations in p53 may lead to genomic instability *in vitro*, with resistant clones arising more rapidly in these tumors, even though the initial response to therapy is the same as that in tumors without p53 mutations. It is also possible that other tumor features that predict a worse prognosis are associated with p53 mutations and influence survival without affecting response to therapy.

In this study p53 over-expression, as determined by IHC, was associated with decreased survival, but p53 mutations identified by sequence analysis were not. Explanations include the possibility that certain p53 mutations have little effect on the tumor phenotype, whereas the presence of p53 stabilization is a surrogate for a mutation with a functional effect. The study was not sufficiently powered to allow for a rigorous examination of the interaction between mutation type and clinical outcome. Furthermore, previously described p53 mutations were not detected in approximately one-quarter of cases in which the IHC assay for p53 was positive. We used stringent criteria for the classification of mutations; only those included in the p53 mutation database maintained at Hôpital Necker-Enfants Malades were considered true mutations. Furthermore, it is possible that some of the mutations identified by sequencing but not included in the database represent true functional mutations.

Large, population-based studies have documented racial disparities in breast cancer outcomes [43]. As demonstrated by Polite and coworkers [44], self-reported African-American women from CALGB 9342 had decreased survival as compared with their Caucasian counterparts, even with adjustment for other factors. In this biomarker substudy, we observed the association between African-American race and worse outcome. A potential explanation for these findings is the observation that these patients were twice as likely to have tumors that were negative for ER, PR and HER2, and triple-negative status was itself associated with significantly poorer OS. Because we did not collect data on post-study treatment, we cannot rule out differences in care received after the study as a cause of the observed disparity. However, our results extend the observations of Olopade [36] and Carey [35] and their groups, who reported that basal-like tumors are more common in women of African ancestry. Because the basal-like phenotype has been associated with a poor prognosis, our findings suggest that differences in the biologic features of tumors may explain the decreased survival of African-American women in our study. To our knowledge, this is the first study to demonstrate that the association between a poor prognosis and the triple-negative phenotype persists in women with metastatic breast cancer.

The study had several limitations. First, we were able to obtain adequate tumor blocks from only approximately one-third of patients enrolled in CALGB 9342. As a result, the power to detect all but substantial differences in outcomes based on biomarkers was limited. Second, we cannot rule out the possibility that the dose-response curve for paclitaxel differs according to HER2 status; unfortunately, our sample was not large enough to carry out such an analysis. Third, 90% of the biomarker assays were performed on blocks from primary tumors (and 10% on blocks from synchronous lymph-node metastases), on the assumption that HER2 status is stable over time regardless of disease progression or interim therapies. A recent abstract has questioned this assumption [45], reporting discordance in HER2 expression between primary and metastatic sites in eight (14%) of 58 patients. Finally, patterns of gene expression may ultimately be more useful than single gene markers in predicting the response to therapy [46-48].

## Conclusion

Molecular subtyping has become commonplace in breast cancer, but the implications of molecular markers in patients with metastatic disease remains unclear. This study suggests that tumor response is not dictated by p53, HER2 or ER status, but that these tumor features impact on tumor regrowth and death in patients with metastatic breast cancer. In addition, the recent observation of increased frequency of 'triple-negative' phenotype in African-American patients was confirmed. Furthermore, this study is the first to point out that the negative prognosis of triple-negative tumors continues beyond the diagnosis of metastasis, but it does not seem to relate to tumor response, at least to paclitaxel. These observations further point out the importance of tumor biology in behavior of breast cancer in the clinical setting.

## Abbreviations

CALGB – Cancer and Leukemia Group B; CI = confidence interval; ER = estrogen receptor; FISH = fluorescent *in situ *hybridization; HER = human epidermal growth factor receptor; IHC = immunohistochemistry; NSABP = National Surgical Adjuvant Breast and Bowel Project; OS = overall survival; PR = progesterone receptor; TTF = time to treatment failure.

## Competing interests

The authors declare that they have no competing interests.

## Authors' contributions

LH conceived the study, supervised the conduct of the project, carried out the molecular genetic studies, and helped to draft the manuscript. GB participated in the design of the study and performed the statistical analysis. NL participated in data analysis and drafted the manuscript. AM conducted the sequencing and analysis of p53. SS performed and analyzed IHC for HER2. DC performed FISH analysis for HER2. MK performed data collection and analysis. IB performed quality assurance of tumor histology and scored HER2 IHC by CB11. DB oversaw statistical design and analysis of the project. ME read and revised various drafts of the manuscript. DH helped to conceive and design the study and revised drafts of the manuscript. EW read and revised various drafts of the manuscript. LD helped design the study and supervised the analysis of tumor specimens for HER2 (FISH and IHC) and p53 (IHC). All authors read and approved the final manuscript.

## Figures and Tables

**Table 1:** Patient characteristics

Characteristic	Patients with biomarker measurements	Patients without biomarker measurements	*P *value
ER positive and/or PR positive (*n *[%])	85 (58%)	159 (62%)	0.45^a^
Prior adjuvant chemotherapy (*n *[%])	98 (60%)	161 (56%)	0.31^a^
Objective response (complete or partial; *n *[%])	38 (24%)	66 (23%)	0.88^a^
Median age (years)	54.9	57.2	0.25^b^
Median follow up (years)	8.3	8.5	0.77^b^
Median number of metastatic sites (*n*)	1	1	0.14^b^
Median disease-free survival (months)	19	31	0.0003^b^
Median time to progression (months)	4.1	4.3	0.62^c^
Median overall survival (months)	12.6	12.3	0.81^c^

Note that data regarding hormone receptor status and prior adjuvant chemotherapy were not available for all patients enrolled in CALGB 9342, and the total number is therefore less than 469 for some of the comparisons. ^a^*P *value is based on the test for comparing two proportions. ^b^*P *value is based on the Wilcoxon rank-sum test for comparing median values. ^c^*P *value is based on a comparison of Kaplan-Meier curves with the log-rank test.

**Table 2:** Results of HER2 Immunohistochemistry and FISH

Assay	Patients (*n *[%])
HER2 CB11 score	
Negative	130 (80%)
Positive	32 (20%)
Total	162 (100%)
HER2 HercepTest score	
0	63 (40%)
1	45 (28%)
2	17 (11%)
3	33 (21%)
Total	158 (100%)
HER2 FISH	
HER2/CEP17 ratio <2.0	113 (74%)
HER2/CEP17 ratio ≥2.0	39 (26%)
Total	152 (100%)

FISH, fluorescent *in situ *hybridization.

**Table 3:** Agreement among methods for measuring HER2

Method	Cohen's kappa	Sensitivity	Specificity
FISH versus CB11	83.0% (SE 5.3%)	97%	93%
HercepTest versus FISH			
(0–1 versus 2–3) versus FISH	72.0% (SE 6.2%)	92%	87%
(0–2 versus 3) versus FISH	79.2% (SE 6.0%)	78%	97%
HercepTest versus CB11			
(0–1 versus 2–3) versus CB11	70.0% (SE 6.3%)	100%	85%
(0–2 versus 3) versus CB11	84.2% (SE 5.4%)	90%	96%

FISH, fluorescent *in situ *hybridization; SE, standard error for Cohen's kappa.

**Table 4:** Objective response rates by clinical characteristics, HER2 status, and p53 status

Variable	Number of patients^a^	Response rate (%)	*P *value^b^
Age			0.045
>50 years	108	29	
≤50 years	50	14	
Performance status			0.81
0	77	23	
1 or 2	80	25	
ER			0.056
Positive	70	17	
Negative	71	31	
PR			0.51
Positive	64	22	
Negative	71	27	
ER/PR			0.22
At least 1 positive	81	20	
Both negative	59	29	
Number of metastatic sites			0.50
0–2	139	25	
>2	17	18	
Disease-free interval			0.10
≤2 years	101	20	
>2 years	57	32	
Prior adjuvant chemotherapy			0.83
Yes	94	24	
No	61	23	
HER2 CBll			0.96
Positive	30	23	
Negative	126	24	
HER2 FISH			0.70
Positive	37	22	
Negative	109	25	
HER2 HercepTest			0.026
Positive: 2–3	46	35	
Negative: 0–1	105	18	
HER2 HercepTest			0.98
Positive: 3	30	23	
Negative: 0–2	121	23	
p53 IHC			0.79
Positive	56	23	
Negative	89	21	
p53 Mutation			0.55
Present	42	21	
Absent	103	26	

^a^Not all patients could be evaluated for a response, and those who could not be evaluated have been excluded. ^b^*P *values are for comparisons of the proportion of patients with a response to paclitaxel in each group. ER, estrogen receptor; FISH, fluorescent *in situ *hybridization; IHC, immunohistochemistry; PR, progesterone receptor.

**Table 5:** Cox proportional hazards univariate modeling for time to treatment failure and overall survival

Variable	Time to treatment failure		Overall survival	
	
	Hazard ratio (95% CI)	*P *value^a^	Hazard ratio (95% CI)	*P *value^a^
Univariate modeling				
Age >50 years	0.59 (0.42–0.83)	0.0051	0.64 (0.46–0.89)	0.041
Performance status: 0 versus 1 or 2	1.30 (0.95–1.78)	0.11	1.41 (1.03–1.94)	0.031
ER positive	0.78 (0.56–1.08)	0.13	0.61 (0.43–0.84)	0.0029
PR positive	0.72 (0.51–1.00)	0.53	0.71 (0.51–1.00)	0.049
ER/PR positive	0.72 (0.51–1.00)	0.53	0.62 (0.44–0.86)	0.0048
Number of metastatic sites: 0 to 2 versus 3+	1.72 (1.03–2.88)	0.37	1.10 (0.66–1.82)	0.72
Disease-free interval: ≤2 years versus >2 years	0.72 (0.52–1.00)	0.49	0.81 (0.59–1.12)	0.20
Prior adjuvant chemotherapy	0.87 (0.63–1.20)	0.39	0.93 (0.68–1.29)	0.67
HER2 positive by CB11	1.44 (0.97–2.15)	0.68	1.34 (0.91–1.99)	0.41
HER2 positive by FISH	1.22 (0.85–1.76)	0.29	1.24 (0.86–1.79)	0.25
HER2 by HercepTest: 0–1 versus 2–3	1.02 (0.73–1.43)	0.90	1.04 (0.74–1.46)	0.83
HER2 by HercepTest: 0–2 versus 3	1.34 (0.91–1.98)	0.14	1.11 (0.76–1.64)	0.59
Multivariate modeling				
Age >50 years	0.99 (0.97–1.00)	0.045	0.99 (0.97–1.00)	0.10
ER/PR positive	1.28 (0.89–1.82)	0.18	1.55 (1.08–2.22)	0.017
HER2 negative on HercepTest: 0–1 versus 2–3	0.97 (0.68–1.40)	0.88	0.93 (0.65–1.35)	0.71

^a^*P *values were calculated using the log-rank test. CI, confidence interval; ER, estrogen receptor; FISH, fluorescence *in situ *hybridization; PR, progesterone receptor.

**Table 6:** HER2 status and median overall survival

Method for ascertaining HER2	HER2 positive	HER2 negative	Log-rank *P *value
CB11	11.3 months	13.1 months	0.14
FISH	10.9 months	13.1 months	0.26
HercepTest: 2–3 versus 0–1	11.5 months	13.2 months	0.84

FISH, fluorescence *in situ *hybridization.

**Table 7:** Race according to biomarkers

Biomarker	Race		*P *value^a^
		
	Caucasian	African-American	
ER positive	58/109 (53%)	13/32 (41%)	0.21
HER2 positive	27/121 (22%)	3/34 (9%)	0.08
Triple negative	26/121 (21%)	16/34 (47%)	0.003

^a^*P *values were obtained using a test of proportions. ER, estrogen receptor.

## References

[B1] Henderson IC, Berry DA, Demetri GD, Cirrincione CT, Goldstein LJ, Martino S, Ingle JN, Cooper MR, Hayes DF, Tkaczuk KH (2003). Improved outcomes from adding sequential paclitaxel but not from escalating doxorubicin dose in an adjuvant chemotherapy regimen for patients with node-positive primary breast cancer. J Clin Oncol.

[B2] Mamounas EP, Bryant J, Lembersky B, Fehrenbacher L, Sedlacek SM, Fisher B, Wickerham DL, Yothers G, Soran A, Wolmark N (2005). Paclitaxel after doxorubicin plus cyclophosphamide as adjuvant chemotherapy for node-positive breast cancer: results from NSABP B-28. J Clin Oncol.

[B3] Reichman BS, Seidman AD, Crown JP, Heelan R, Hakes TB, Lebwohl DE, Gilewski TA, Surbone A, Currie V, Hudis CA (1993). Paclitaxel and recombinant human granulocyte colony-stimulating factor as initial chemotherapy for metastatic breast cancer. J Clin Oncol.

[B4] Seidman AD, Tiersten A, Hudis C, Gollub M, Barrett S, Yao TJ, Lepore J, Gilewski T, Currie V, Crown J (1995). Phase II trial of paclitaxel by 3-hour infusion as initial and salvage chemotherapy for metastatic breast cancer. J Clin Oncol.

[B5] Nabholtz JM, Gelmon K, Bontenbal M, Spielmann M, Catimel G, Conte P, Klaassen U, Namer M, Bonneterre J, Fumoleau P, Winograd B (1996). Multicenter, randomized comparative study of two doses of paclitaxel in patients with metastatic breast cancer. J Clin Oncol.

[B6] Paridaens R, Biganzoli L, Bruning P, Klijn JG, Gamucci T, Houston S, Coleman R, Schachter J, Van Vreckem A, Sylvester R (2000). Paclitaxel versus doxorubicin as first-line single-agent chemotherapy for metastatic breast cancer: a European Organization for Research and Treatment of Cancer Randomized Study with cross-over. J Clin Oncol.

[B7] Sledge GW, Neuberg D, Bernardo P, Ingle JN, Martino S, Rowinsky EK, Wood WC (2003). Phase III trial of doxorubicin, paclitaxel, and the combination of doxorubicin and paclitaxel as front-line chemotherapy for metastatic breast cancer: an intergroup trial (E1193). J Clin Oncol.

[B8] Winer EP, Berry DA, Woolf S, Duggan D, Kornblith A, Harris LN, Michaelson RA, Kirshner JA, Fleming GF, Perry MC (2004). Failure of higher-dose paclitaxel to improve outcome in patients with metastatic breast cancer: cancer and leukemia group B trial 9342. J Clin Oncol.

[B9] Slamon DJ, Clark GM, Wong SG, Levin WJ, Ullrich A, McGuire WL (1987). Human breast cancer: correlation of relapse and survival with amplification of the HER-2/neu oncogene. Science.

[B10] Thor AD, Berry DA, Budman DR, Muss HB, Kute T, Henderson IC, Barcos M, Cirrincione C, Edgerton S, Allred C (1998). erbB-2, p53, and efficacy of adjuvant therapy in lymph node-positive breast cancer. J Natl Cancer Inst.

[B11] Paik S, Bryant J, Park C, Fisher B, Tan-Chiu E, Hyams D, Fisher ER, Lippman ME, Wickerham DL, Wolmark N (1998). erbB-2 and response to doxorubicin in patients with axillary lymph node-positive, hormone receptor-negative breast cancer. J Natl Cancer Inst.

[B12] Montgomery RB, Guzman J, O'Rourke DM, Stahl WL (2000). Expression of oncogenic epidermal growth factor receptor family kinases induces paclitaxel resistance and alters beta-tubulin isotype expression. J Biol Chem.

[B13] Yu D, Jing T, Liu B, Yao J, Tan M, McDonnell TJ, Hung MC (1998). Overexpression of ErbB2 blocks Taxol-induced apoptosis by upregulation of p21Cip1, which inhibits p34Cdc2 kinase. Mol Cell.

[B14] Knuefermann C, Lu Y, Liu B, Jin W, Liang K, Wu L, Schmidt M, Mills GB, Mendelsohn J, Fan Z (2003). HER2/PI-3K/Akt activation leads to a multidrug resistance in human breast adenocarcinoma cells. Oncogene.

[B15] Pegram MD, Finn RS, Arzoo K, Beryt M, Pietras RJ, Slamon DJ (1997). The effect of HER-2/neu overexpression on chemotherapeutic drug sensitivity in human breast and ovarian cancer cells. Oncogene.

[B16] Baselga J, Seidman AD, Rosen PP, Norton L (1997). HER2 overexpression and paclitaxel sensitivity in breast cancer: therapeutic implications. Oncology (Williston Park).

[B17] Van Poznak C, Tan L, Panageas KS, Arroyo CD, Hudis C, Norton L, Seidman AD (2002). Assessment of molecular markers of clinical sensitivity to single-agent taxane therapy for metastatic breast cancer. J Clin Oncol.

[B18] Hamilton A, Larsimont D, Paridaens R, Drijkoningen M, van de Vijver M, Bruning P, Hanby A, Houston S, Treilleux I, Guastalla JP (2000). A study of the value of p53, HER2, and Bcl-2 in the prediction of response to doxorubicin and paclitaxel as single agents in metastatic breast cancer: a companion study to EORTC 10923. Clin Breast Cancer.

[B19] Colomer R, Montero S, Lluch A, Ojeda B, Barnadas A, Casado A, Massuti B, Cortes-Funes H, Lloveras B (2000). Circulating HER2 extracellular domain and resistance to chemotherapy in advanced breast cancer. Clin Cancer Res.

[B20] Gianni L (1997). Future directions of paclitaxel-based therapy of breast cancer. Semin Oncol.

[B21] Konecny GE, Thomssen C, Luck HJ, Untch M, Wang HJ, Kuhn W, Eidtmann H, du Bois A, Olbricht S, Steinfeld D (2004). Her-2/neu gene amplification and response to paclitaxel in patients with metastatic breast cancer. J Natl Cancer Inst.

[B22] Muller V, Witzel I, Luck HJ, Kohler G, von Minckwitz G, Mobus V, Sattler D, Wilczak W, Loning T, Janicke F (2004). Prognostic and predictive impact of the HER-2/neu extracellular domain (ECD) in the serum of patients treated with chemotherapy for metastatic breast cancer. Breast Cancer Res Treat.

[B23] Pharoah PD, Day NE, Caldas C (1999). Somatic mutations in the p53 gene and prognosis in breast cancer: a meta-analysis. Br J Cancer.

[B24] Alsner J, Yilmaz M, Guldberg P, Hansen LL, Overgaard J (2000). Heterogeneity in the clinical phenotype of TP53 mutations in breast cancer patients. Clin Cancer Res.

[B25] Wahl AF, Donaldson KL, Fairchild C, Lee FY, Foster SA, Demers GW, Galloway DA (1996). Loss of normal p53 function confers sensitization to Taxol by increasing G2/M arrest and apoptosis. Nat Med.

[B26] Zhang CC, Yang JM, White E, Murphy M, Levine A, Hait WN (1998). The role of MAP4 expression in the sensitivity to paclitaxel and resistance to vinca alkaloids in p53 mutant cells. Oncogene.

[B27] Zhang CC, Yang JM, Bash-Babula J, White E, Murphy M, Levine AJ, Hait WN (1999). DNA damage increases sensitivity to vinca alkaloids and decreases sensitivity to taxanes through p53-dependent repression of microtubule-associated protein 4. Cancer Res.

[B28] Strobel T, Kraeft SK, Chen LB, Cannistra SA (1998). BAX expression is associated with enhanced intracellular accumulation of paclitaxel: a novel role for BAX during chemotherapy-induced cell death. Cancer Res.

[B29] Gualberto A, Aldape K, Kozakiewicz K, Tlsty TD (1998). An oncogenic form of p53 confers a dominant, gain-of-function phenotype that disrupts spindle checkpoint control. Proc Natl Acad Sci USA.

[B30] Perou CM, Sorlie T, Eisen MB, van de Rijn M, Jeffrey SS, Rees CA, Pollack JR, Ross DT, Johnsen H, Akslen LA (2000). Molecular portraits of human breast tumours. Nature.

[B31] Gruvberger S, Ringner M, Chen Y, Panavally S, Saal LH, Borg A, Ferno M, Peterson C, Meltzer PS (2001). Estrogen receptor status in breast cancer is associated with remarkably distinct gene expression patterns. Cancer Res.

[B32] van 't Veer LJ, Dai H, van de Vijver MJ, He YD, Hart AA, Mao M, Peterse HL, van der Kooy K, Marton MJ, Witteveen AT (2002). Gene expression profiling predicts clinical outcome of breast cancer. Nature.

[B33] West M, Blanchette C, Dressman H, Huang E, Ishida S, Spang R, Zuzan H, Olson JA, Marks JR, Nevins JR (2001). Predicting the clinical status of human breast cancer by using gene expression profiles. Proc Natl Acad Sci USA.

[B34] Sorlie T, Perou CM, Tibshirani R, Aas T, Geisler S, Johnsen H, Hastie T, Eisen MB, van de Rijn M, Jeffrey SS (2001). Gene expression patterns of breast carcinomas distinguish tumor subclasses with clinical implications. Proc Natl Acad Sci USA.

[B35] Carey LA, Perou CM, Livasy CA, Dressler LG, Cowan D, Conway K, Karaca G, Troester MA, Tse CK, Edmiston S (2006). Race, breast cancer subtypes, and survival in the Carolina Breast Cancer Study. JAMA.

[B36] Livasy CA, Karaca G, Nanda R, Tretiakova MS, Olopade OI, Moore DT, Perou CM (2006). Phenotypic evaluation of the basal-like subtype of invasive breast carcinoma. Mod Pathol.

[B37] Wang ZC, Lin M, Wei LJ, Li C, Miron A, Lodeiro G, Harris L, Ramaswamy S, Tanenbaum DM, Meyerson M (2004). Loss of heterozygosity and its correlation with expression profiles in subclasses of invasive breast cancers. Cancer Res.

[B38] Universal Mutation Database http://www.umd.necker.fr/.

[B39] Norberg T, Lennerstrand J, Inganas M, Bergh J (1998). Comparison between p53 protein measurements using the luminometric immunoassay and immunohistochemistry with detection of p53 gene mutations using cDNA sequencing in human breast tumors. Int J Cancer.

[B40] McShane LM, Altman DG, Sauerbrei W, Taube SE, Gion M, Clark GM (2005). Reporting recommendations for tumor marker prognostic studies (REMARK). J Natl Cancer Inst.

[B41] Henderson IC NL, Berry DA (2003). In reply: Benefit of paclitaxel in estrogen receptor-negative versus estrogen receptor-positive early breast cancer [letter]. J Clin Oncol.

[B42] Martin M, Pienkowski T, Mackey J, Pawlicki M, Guastalla JP, Weaver C, Tomiak E, Al-Tweigeri T, Chap L, Juhos E (2005). Adjuvant docetaxel for node-positive breast cancer. N Engl J Med.

[B43] Jemal A, Tiwari RC, Murray T, Ghafoor A, Samuels A, Ward E, Feuer EJ, Thun MJ (2004). Cancer statistics, 2004. CA Cancer J Clin.

[B44] Polite BN CC, Fleming GF, Berry DA, Seidman AD, Muss H, Norton L, Hudis C, Winer EP (2005). Understanding racial differences in outcome from metastatic breast cancer (MBC): A pooled analysis of Cancer and Leukemia Group B (CALGB) 9342 and 9840 [abstract 3097]. Breast Cancer Res Treat.

[B45] Zidan J, Dashkovsky I, Stayerman C, Basher W, Cozacov C, Hadary A (2005). Comparison of HER-2 overexpression in primary breast cancer and metastatic sites and its effect on biological targeting therapy of metastatic disease. Br J Cancer.

[B46] Chang JC, Wooten EC, Tsimelzon A, Hilsenbeck SG, Gutierrez MC, Elledge R, Mohsin S, Osborne CK, Chamness GC, Allred DC, O'Connell P (2003). Gene expression profiling for the prediction of therapeutic response to docetaxel in patients with breast cancer. Lancet.

[B47] Rouzier R, Rajan R, Wagner P, Hess KR, Gold DL, Stec J, Ayers M, Ross JS, Zhang P, Buchholz TA (2005). Microtubule-associated protein tau: a marker of paclitaxel sensitivity in breast cancer. Proc Natl Acad Sci USA.

[B48] Harris LN, Perou C, Szallasi Z, Eklund A, Carter S, You F, Broadwater G, Monovich L, Winer E, Erlander M, Ellis M (2005). Microarray profiling is feasible using archived tissue from a Cooperative Group Clinical Trial: results from a pilot study in CALGB 9342. ASCO Meeting Abstracts.

